# Supplementary material for: Identification of lead vacancy defects in lead halide perovskites
Source: Nat Commun. 2021 Sep 22;12:5566. doi: 10.1038/s41467-021-25937-1 (PMC8458286; doi:10.1038/s41467-021-25937-1)
Supplement: Supplementary file 1 — Supplementary Information [file 41467_2021_25937_MOESM1_ESM.pdf]

Supplementary Information

Identification of lead vacancy defects in lead halide perovskites

David J. Keeble, Julia Wiktor, Sandeep K. Pathak, Laurie J. Phillips, Marcel Dickmann, Ken Durose, Henry J. Snaith and Werner Egger

Supplementary Note 1: Theoretical calculations

While the calculation values reported in manuscript were performed using semilocal GGA functionals and neglect the spin-orbit coupling (SOC) it is known that using hybrid functionals and inclusion of SOC can affect the defect calculations in halide perovskites significantly[1]. It is normally of particular importance to use hybrid functionals and include SOC when studying defects in charge states that differ from their formal value (*i.e.* their usual oxidation states). By contrast, when the defects are in their usual oxidation states, *e.g.* V_{Pb}^{2-} or V_{MA}^{-}, as is the case here the resulting defect geometries are not significantly altered. Nevertheless, a series of calculations were performed for the V_{Pb}^{2-} defect using Vienna *ab initio* package [2, 3] to determine the possible effects on the resulting positron lifetime values. Calculations were performed relaxing the defect using three different levels of theory, firstly using the GGA functional and neglecting SOC, then including SOC, and finally using the hybrid PBE0 functional and including SOC. In each case the resulting geometry was used to calculate positron lifetime using ABINIT. It was found that using GGA and including SOC resulted in a reduction of the calculated positron lifetime by 6 ps, however, using the hybrid functionals and including SOC increased this value by 5 ps. We therefore conclude that the effects on the computed positron lifetime value resulting from the changing both the functional and including SOC largely cancel each other, and the approach used in the calculations is therefore valid.

Supplementary Note 2: Further information on $MAPbI_3$ Positron Annihilation Lifetime Spectroscopy (PALS) experiments and results

The deconvolution results for the positron lifetime spectra measured from the single crystal using implantation energies of 4 keV and 16 keV are given in Supplementary Table 1. The Markovian positron implantation profiles for 4 keV and 16 keV for $MAPbI_3$ are shown in Supplementary Figure 1.

Supplementary Table 1. Experimental positron lifetime component results for $MAPbI_3$ single crystal sample. Deconvolved lifetime component values and intensities for positron implantation energy E (keV).

E [keV]	χ^2	τ_1 [ps]	I_1 [%]	τ_2 [ps]	I_2 [%]	τ_3 [ns]	I_3 [%]	τ_4 [ns]	I_4 [%]	τ_{ave} [ps]	κ_D [s^{-1}]
4	1.083	42(10)	4.6(5)	368(4)	94(2)	0.6(3)	1.4(2.5)	2.8(1.3)	0.07(5)	358(2)	2.0(5)E10
16	1.205	151(28)	3.9(9)	369(2)	96(1)	1.1(2)	0.4(1)			363(1)	3.8(8)E9

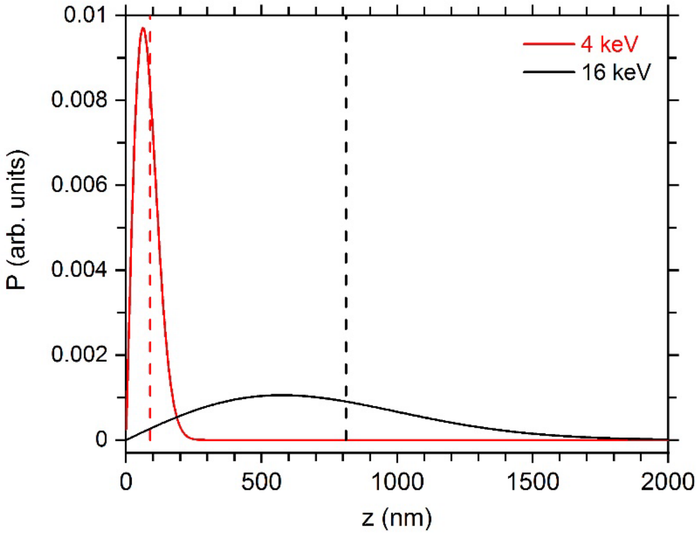

Supplementary Fig. 1. Markovian positron implantation depth profiles. The mean depths are shown with a dashed line.

The results from the University of Oxford film measured at 2 keV and 4 keV are given in Supplementary Table 2, and the results for the three University of Liverpool films measured at

implantation energies of 2.5 keV and 4 keV are shown in Supplementary Tables 3 -5 (Note the relevant Markovian positron implantation profiles are shown in Fig. 3(b) in the main manuscript).

Supplementary Table 2. Experimental positron lifetime component results for University of Oxford $MAPbI_3$ thin film. Deconvolved lifetime component values and intensities for positron implantation energy E (keV).

E [keV]	χ^2	τ_1 [ps]	I_1 [%]	τ_2 [ps]	I_2 [%]	τ_3 [ns]	I_3 [%]	τ_4 [ns]	I_4 [%]	τ_{ave} [ps]	κ_D [s^{-1}]
2	1.010	119(45)	2.2(8)	371(5)	94(1)	0.7(1)	3.5(1.6)	2.9(4)	0.35(7)	387(1)	5.6(2.2)E9
4	1.063	151(54)	3(2)	367(7)	92(1)	0.8(1)	5(1)	2.4(3)	0.3(3)	390(1)	3.8(1.5)E9

Supplementary Table 3. Experimental positron lifetime component results for University of Liverpool $MAPbI_3$ thin film. Deconvolved lifetime component values and intensities for positron implantation energy E (keV).

E [keV]	χ^2	τ_1 [ps]	I_1 [%]	τ_2 [ps]	I_2 [%]	τ_3 [ns]	I_3 [%]	τ_{ave} [ps]	κ_D [s^{-1}]
2.5	0.979	109(19)	2.7(2)	375(1)	97.3(2)	2.3(4)	0.05(1)	369(1)	6.3(1.1)E9
4	1.028	76(9)	3.7(2)	375(1)	96.3(2)	1.9(1)	0.09(2)	365(1)	1.0(2)E10

Supplementary Table 4. Experimental positron lifetime component results for University of Liverpool $MAPbI_3$ thin film (Anneal A). Deconvolved lifetime component values and intensities for positron implantation energy E (keV).

E [keV]	χ^2	τ_1 [ps]	I_1 [%]	τ_2 [ps]	I_2 [%]	τ_3 [ns]	I_3 [%]	τ_{ave} [ps]	κ_D [s^{-1}]
2.5	1.092	97(15)	2.3(2)	370(1)	98(1)	1.7(1)	0.28(2)	368(1)	7.5(1.2)E9
4	1.111	75(11)	3.1(2)	369(1)	96.2(2)	1.25(4)	0.73(5)	367(1)	1.0(2)E10

Supplementary Table 5. Experimental positron lifetime component results for University of Liverpool $MAPbI_3$ thin film (Anneal B). Deconvolved lifetime component values and intensities for positron implantation energy E (keV).

E [keV]	χ^2	τ_1 [ps]	I_1 [%]	τ_2 [ps]	I_2 [%]	τ_3 [ns]	I_3 [%]	τ_{ave} [ps]	κ_D [s^{-1}]
2.5	1.027	49(27)	2(1)	367(1)	98(1)	1.4(1)	0.29(3)	364(3)	1.7(9)E10
4	1.067	105(13)	3.1(2)	369(1)	96.2(3)	1.32(5)	0.54(5)	366(1)	6.6(9)E9

It should be noted that PALS measurements were performed on an Oxford sample which had an additional PMMA environmental protection layer deposited on the $MAPbI_3$ film. These results are described in a following section.

The defect trapping rates given in Supplementary Tables 1 - 5 have been determined with Eq. 2 using the first two experimental lifetime component lifetime and intensity values. The positronium component (see relevant following section) intensities have been subtracted and the positron annihilation component intensities renormalized.

Considering the dominant defect lifetime component, the intensity of this component is 92% or greater in all the spectra measured from the five samples. The average value of this component, obtained from all the spectra, yields the value 370(3) ps. One thin film sample returned a value of 375(1) ps for the defect component, the other samples yield an average value of 368.8(1.4) ps.

The timing calibration of the spectrometer typically gives an uncertainty in the time per channel of the multichannel analyzer of ~0.3 %, or a typical possible timing uncertainty of ~1 ps for the range of lifetimes of interest.

Supplementary Note 3: Standard Trapping Model defect concentration calculations.

The Standard Trapping Model (STM) predicts that the rate of positron trapping, κ_D, is proportional to vacancy concentration, [V], *i.e.* $\kappa_D = \mu_V[V]$, and the constant of proportionality is the defect specific trapping coefficient, μ_V. Assuming the presence of one positron trapping defect the one

defect STM (1D-STM) predicts two positron lifetime component, a short first lifetime component with a value less than τ_B the bulk, perfect lattice, lifetime for the material and termed the reduced bulk lifetime component, τ_{RB}, with intensity I_{RB} and a second lifetime component having a value characteristic of the vacancy defect, τ_D, and intensity I_D [4]. The bulk lifetime may be calculated from the experimentally determined lifetime component values,

$$\tau_B = \left(\frac{I_{RB}}{\tau_{RB}} - \frac{I_D}{\tau_D} \right)^{-1} \qquad (1).$$

The rate of positron trapping to the defect, κ_D, may be determined using,

$$\kappa_D = \frac{I_D}{I_{RB}} \left(\frac{1}{\tau_B} - \frac{1}{\tau_D} \right) = I_D \left(\frac{1}{\tau_{RB}} - \frac{1}{\tau_D} \right) \qquad (2).$$

The deconvolved lifetime results given in Supplementary Tables 1 – 5 clearly demonstrate that positron trapping is dominated by a single positron trapping defect with a lifetime value in the range 367 – 375 ps. A small fraction of positrons (≤ 5 %) form positronium (see below) and can be excluded from the analysis.

If the experimental lifetime component values are used to calculate the 1D-STM bulk lifetime using Eq. 1 then an average value of 337(21) ps is obtained from the ten spectra. However, the reliability of the deconvolution in determining lifetime components with values less than approximately 50 ps is degraded, and so if 4 keV spectrum fit from the crystalline sample (Supplementary Table 1) is excluded for that reason, an average value for the calculated bulk lifetime of 342(12) ps results.

An approximate vacancy defect concentration, [V], can be estimated from the experimental defect positron rates as $[\mathrm{V}] = \kappa_D / \mu_\mathrm{V}$. The values for defect specific trapping coefficient, μ_V, for negatively charged vacancy defects in various semiconductors have been reported to be in the range[4] ~ 1 x 10^{15} – 3 x 10^{16} s^{-1}. Using the highest estimated defect specific trapping rate, and expressing this rate in terms of defect density by using the density of lattice sites for $MAPbI_3$ (2.04 x 10^{22} cm^{-3}) the lowest vacancy defect density value obtained is 2.6 x 10^{15} cm^3. This provides the lowest plausible estimate of a lead vacancy related defect concentration observed in these measurements. Using all the positron trapping rates given in Supplementary Tables 1 – 5 an average value of 5.1(1.5) x 10^{15} cm^3 is then obtained. However, if a more typical value for μ_V of 2 x 10^{15} s^{-1} is used an average defect density of 9.3(5.7) x 10^{16} cm^3 is obtained. The defect concentrations can also be reported as the number of defects per atom on the Pb sublattice by using the defect specific trapping rate in the units s^{-1} directly. If this is done then the average defect density obtained using μ_V of 2 x 10^{15} s^{-1} is 4.6(2.8) x 10^{-6} or approximately 5 ppm.

Supplementary Note 4: Positronium formation and annihilation.

In the $MAPbI_3$ films studied almost all implanted positrons annihilate directly with electrons, however, in some samples a small fraction form positronium (Ps) prior to annihilation, the bound state of a positron – electron pair. Positronium normally forms in insulators in association with open-volume defects of sufficient size, the process can also occur at surfaces. Positronium forms either in a spin singlet state, para-positronium (*p*-Ps), or in the triplet state, ortho-positronium (*o*-Ps). Para-positronium decays with a lifetime of approximately 125 ps while, in stark contrast, *o*-Ps has a lifetime of 142 ns in vacuum[4]. When *o*-Ps forms within a confining within a nanovoid it will annihilate via a pick-off process with a lifetime in the range $\sim 0.5 < \tau_{o-\mathrm{Ps}} < 142$ ns, dependent

on the size of the open volume. The size of the open volume can be inferred from using a simple quantum mechanical model, the Tao-Eldrup model, but this model must be modified for void sizes greater than approximately 6 nm. The model also breaks down for small open volume sizes which predict *o*-Ps lifetimes less than 1 ns [5]. In consequence, it is currently not possible to reliable attribute lifetimes in 600 – 800 ps range observed here from one of the thin film samples, and from the near surface region of the single crystal (Supplementary Tables 1 and 2).

Supplementary Note 5: PMMA encapsulated $MAPbI_3$ film PALS results.

$MAPbI_3$ samples were loaded into the high vacuum sample chamber rapidly, but nevertheless were exposed to the ambient environment for one to two minutes. However, the $MAPbI_3$ films from University of Oxford included a sample with an additional thin, approximately 100 nm, PMMA protective encapsulation layer. For an implantation energy of 1 keV positrons implant exclusively in the PMMA capping layer (Supplementary Figure 2). The spectrum deconvolution results are shown in Supplementary Table 6. The characteristic, prominent, nanosecond third lifetime component results from pick-off annihilation of ortho-positronium (*o*-Ps) localized in open-volume within the polymer. The first lifetime component contains the contribution from direct annihilation of para-positronium, and the second lifetime component results from positrons that do not form positronium and annihilate from the amorphous matrix. Importantly, at 4 keV more than 85% of the positrons are implanted into, and annihilating from, the $MAPbI_3$ film. The intensity of the nanosecond *o*-Ps component resulting from annihilation events within the PMMA layer is markedly reduced. The most intense component has a lifetime of 374 ps (I = 91%), in agreement with the lifetime value for the dominant lifetime component observed from $MAPbI_3$ samples without the PMMA encapsulating layer (Supplementary Table 1 – 5).

Supplementary Table 6. Experimental positron lifetime component results for University of Oxford $MAPbI_3$ thin film with PMMA encapsulation layer. Deconvolved lifetime component values and intensities for positron implantation energy E (keV).

E [keV]	χ^2	τ_1 [ps]	I_1 [%]	τ_2 [ps]	I_2 [%]	τ_3 [ns]	I_3 [%]	τ_{ave} [ps]
1	1.145	215(7)	23(2)	429(5)	58(2)	2.10(1)	18.8(1)	695(1)
4	1.130	91(8)	5.5(2)	374(1)	91.3(2)	1.91(2)	3.2(1)	407(1)

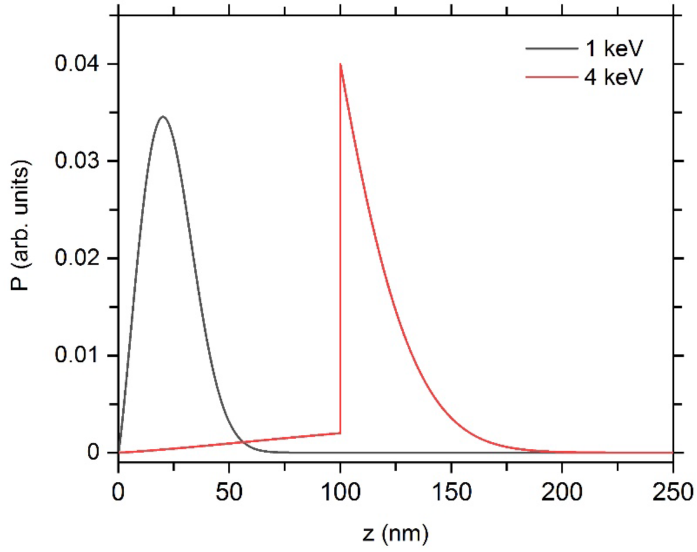

Supplementary Fig. 2. Markovian positron implantation depth profiles 100 nm PMMA on $MAPbI_3$.

References

1. Du M-H. Density Functional Calculations of Native Defects in $CH_3NH_3PbI_3$: Effects of Spin–Orbit Coupling and Self-Interaction Error. *J Phys Chem Lett* **6**, 1461-1466 (2015).
2. Kresse G, Furthmuller J. Efficient iterative schemes for ab initio total-energy calculations using a plane-wave basis set. *Phys Rev B* **54**, 11169-11186 (1996).

3. Kresse G, Hafner J. Ab initio molecular dynamics for liquid metals. *Phys Rev B* **47**, 558-561 (1993).

4. Krause-Rehberg, R, Leipner, HS. *Positron Annihilation in Semiconductors,* Springer-Verlag (1999).

5. Zgardzinska, B. The size of smallest subnanometric voids estimated by positron annihilation method. Correction to the Tao-Eldrup model. *Chem. Phys. Lett.* **2015** *622*, 20.
